# Computational approaches to support comparative analysis of multiparametric tests: Modelling versus Training

**DOI:** 10.1371/journal.pone.0238593

**Published:** 2020-09-03

**Authors:** John M. S. Bartlett, Jane Bayani, Elizabeth N. Kornaga, Patrick Danaher, Cheryl Crozier, Tammy Piper, Cindy Q. Yao, Janet A. Dunn, Paul C. Boutros, Robert C. Stein, OPTIMA Trial Management Group

**Affiliations:** 1 Diagnostic Development, Ontario Institute for Cancer Research, Toronto, Ontario, Canada; 2 Laboratory Medicine and Pathobiology, University of Toronto, Toronto, Ontario, Canada; 3 Edinburgh Cancer Research Centre, Edinburgh, United Kingdom; 4 Tom Baker Cancer Centre, Calgary, Alberta, Canada; 5 Computational Biology Program, Ontario Institute for Cancer Research, Toronto, Ontario, Canada; 6 Warwick Medical School, University of Warwick, Coventry, United Kingdom; 7 Department of Medical Biophysics, University of Toronto, Toronto, Ontario, Canada; 8 Department of Pharmacology & Toxicology, University of Toronto, Toronto, Ontario, Canada; 9 UCL (University College London) and National Institute for Health Research University College London Hospitals Biomedical Research Centre, London, United Kingdom; Gangnam Severance Hospital, Yonsei University College of Medicine, REPUBLIC OF KOREA

## Abstract

Multiparametric assays for risk stratification are widely used in the management of breast cancer, with applications being developed for a number of other cancer settings. Recent data from multiple sources suggests that different tests may provide different risk estimates at the individual patient level. There is an increasing need for robust methods to support cost effective comparisons of test performance in multiple settings. The derivation of similar risk classifications using genes comprising the following multi-parametric tests Oncotype DX^®^ (Genomic Health.), Prosigna^™^ (NanoString Technologies, Inc.), MammaPrint^®^ (Agendia Inc.) was performed using different computational approaches. Results were compared to the actual test results. Two widely used approaches were applied, firstly computational “modelling” of test results using published algorithms and secondly a “training” approach which used reference results from the commercially supplied tests. We demonstrate the potential for errors to arise when using a “modelling” approach without reference to real world test results. Simultaneously we show that a “training” approach can provide a highly cost-effective solution to the development of real-world comparisons between different multigene signatures. Comparisons between existing multiparametric tests is challenging, and evidence on discordance between tests in risk stratification presents further dilemmas. We present an approach, modelled in breast cancer, which can provide health care providers and researchers with the potential to perform robust and meaningful comparisons between multigene tests in a cost-effective manner. We demonstrate that whilst viable estimates of gene signatures can be derived from modelling approaches, in our study using a training approach allowed a close approximation to true signature results.

## Introduction

Multi-parametric molecular tests are now considered essential to the diagnostic management of luminal-type (ER+ve HER2-ve) early breast cancer and are included in key guidelines [1] as a pre-requisite for staging of breast cancer patients, to direct prognostication and to select patients for chemotherapy treatment [2, 3]. Additionally, applications are rapidly being extended into other settings (e.g. early prostate cancer). Two crucial challenges remain. Firstly, the continued appearance of reports highlighting disagreements between tests is disquieting for physicians, health care providers and patients [4, 5], since they raise the question “*did I get the right test*?” Secondly the lack of consistency in the information produced by different tests raises the question “*are two tests better than one*?”–followed immediately by “*which tests and in which order*?” The pivotal role of molecular signatures has been confirmed by recent results from the TAILORx study validating their utility to direct use of chemotherapy [2]. In this context, an error in assigning appropriate risk classifications using molecular signatures could have a significant impact on patient treatment and outcomes. Previous studies comparing *in silico*-generated risk signatures, aimed to recapitulate performance of real-world tests, have been hampered by: a) incomplete gene coverage from some signatures and b) an inability to confirm the accuracy of simulated test results by comparison with data from actual test results [6].

Early reports of disagreements between tests, based on *in silico* analyses of existing RNA array data, were frequently attributed to methodological challenges and incomplete gene coverage [7–11]. Two recent and striking strands of evidence raise significant questions about “*what to do when tests disagree*”. Firstly data from direct comparisons between tests in large clinical trial-derived cohorts provide consistent evidence that combining test results generally improves prognostic value [12, 13], reflecting the relatively modest performance of many current multiparametric tests [14]. Secondly a direct comparison of test results, when tests were performed exactly to standard vendor protocols, demonstrated marked disagreement in both risk categorization and subtyping of cancers between commonly used multiparameter assays [4].

We assessed two approaches to the generation of simulated risk scores leading to the generation of two different simulated results for each of the multi-parametric tests examined. Both methods used data from all the representative genes (both those used for normalization and reporting) for each of the relevant tests. Method 1 focused on computational modelling to allow recapitulation of results using the reported algorithm formulae for the individual assays of the relevant multi-parametric test following a global normalization procedure. The base assumption here was that the algorithms generated by the various authors generating these signatures could be applied across platforms without correction and was specifically chosen as it recapitulates the approaches taken by previous authors [7–11]. Method 2 used a training and validation approach based on results obtained from the OPTIMA prelim study [4]. The method with the best fit to actual results across either the entire OPTIMA prelim cohort (method 1) or the validation set (method 2) would be selected for the comparisons between test performance in the future. We predicted better performance for the “training” versus “modelling” approaches. Our study highlights the importance of ensuring appropriate benchmarking of modelling approaches if downstream analyses comparing test performance is to be informative in the clinical setting.

## Materials and methods

### Patient samples

The OPTIMA (Optimal Personalized Treatment of Early Breast Cancer Using Multiparameter Analysis) trial (ISRCTN 42400492) seeks to identify a method of selection that reduces chemotherapy use for patients with hormone sensitive primary breast cancer without detriment to recurrence and survival, thereby allowing people unlikely to benefit from chemotherapy to avoid unnecessary side effects. OPTIMA is a randomised trial comparing the outcome of patients with high-risk ER+ve HER2-ve disease managed using test-directed assignment to chemotherapy with standard management (chemotherapy) in a non-inferiority design. OPTIMA prelim was the feasibility phase of the study which selected the testing technology to be used in the main trial and demonstrated that the main trial is feasible [15]. Between October 2012 and June 2014, 313 patients were randomly assigned from 35 UK hospitals of whom 302 had samples available for multiparameter testing [4]. Results from multiple molecular tests were available for 274 (87.5%) patients [4]. Ethical approval for the OPTIMA trial was provided by the NHS Health Research Authority, NRES Committee South East Coast—Surrey, reference 12/LO/0515 this specific study was approved by the University of Toronto Research Ethics Board, reference 29510 & 38369.

### RNA extraction and expression profiling using NanoString

Profiling of available samples from the OPTIMA prelim cohort (n = 274) for this study was performed using mRNA previously extracted [4] for the trial, and analysed using the NanoString codeset as described by Bayani et al. [14].

### Derivation of “signature-*like*” and “signature-*trained*” risk stratification scores from candidate assays

We assessed two different approaches to the generation of simulated risk scores leading to the generation of two different simulated results for each test examined. Both methods used data from all the representative genes (both those used for normalization and reporting) for each of the relevant tests. For all comparisons we either used all risk scores as continuous values (regression analyses) or categorized risk scores using accepted approaches defined by each assay [16–18] with one modification as follows: For Oncotype DX scores we used the cut-off of 25 to discriminate between intermediate and high risk scores as previously in the OPTIMA prelim study [4] and as applied to the TAILORx study [2]. Therefore for Prosigna, (ROR-PT) we used the following categories low risk 0–40, intermediate risk 40–60 and high risk >60; for Oncotype DX we used low risk 0–18, intermediate risk 18–25 and high risk >25. These categories apply only to the cross tabulations in Supplementary S1-S6 in S1 File.

#### Modelling approach: “Signature-*like*” risk stratification scores

The method previously reported by us in Bayani et al. [14] used a derivation of risk classifications using all genes comprising the following multi-parametric tests Oncotype DX^®^ (Oncotype DX^®^ (Genomic Health Inc.) [16, 17], Prosigna^™^(NanoString Technologies, Inc.) [19, 20], MammaPrint^®^ (Agendia Inc.) [18]. To generate the “Oncotype DX-*like*” Recurrence Score, NanoString gene expression intensity values were normalized and transformed to fit the measurement range as described previously [14]. Individual recurrence scores were calculated and patients were then classified into high, intermediate or low risk groups based the derived recurrence scores. For the “Prosigna-*like*-Risk of Recurrence Score” (“Prosigna-*like* ROR-PT”), samples were processed based on the method outlined by Parker et al. [21]. For the MammaPrint-*like* Risk Score, samples were scored based on the gene70 function of the genefu R package (v1.14.0). Derivation of low and high-risk categories were modelled according to van de Vijver et al. [19].

This approach (Method 1), resulting in scores we describe as “signature-*like*”, focused on computational recapitulation of risk stratification scores using the published algorithms for the individual assays of the relevant multi-parametric tests following a single global normalization procedure. The base assumption was that the algorithms for generating these signatures could be applied across normalised data from different platforms without correction and was specifically chosen as it recapitulates the approaches taken by previous authors [7–11] who sought to perform between test comparisons. For all tests we used the suffix “*-like*” to discriminate the computationally derived assays scores from the actual assay derived scores, e.g. “Oncotype DX-*like*” vs Oncotype DX^™^.

#### Training approach “Signature-*trained*” risk stratification scores

A second approach (Method 2), resulting in scores termed “signature-*trained*”, used a training and validation approach based on results obtained from the OPTIMA prelim study [4]. Performing analysis of the OPTIMA prelim samples using both commercial assays (OncotypeDX^®^ (Genomic Health Inc.) [16, 17], Prosigna^®^ NanoString Technologies, Inc.) [19, 20], MammaPrint^®^ (Agendia Inc.) [18], and our own RNA profiling results [14] allowed us to use the OPTIMA prelim cohort to train results for these multi-parametric assays. For this approach the OPTIMA prelim data set was split 50:50 into a training and validation set. Results from the training set were used to optimize the fit between signature-*trained* scores and actual signature results from the OPTIMA prelim study. Once solutions were locked (i.e. the optimal fit was achieved), results were validated using the validation cohort from the OPTIMA prelim study. These results (validation set only) were used assess the goodness of fit between the “signature-*trained*” scores and the true scores for each individual assay/score although all results were used in cross tab comparisons (Supplementary Tables in S1 File).

#### a) Generation of Prosigna-*trained* scores

The Prosigna algorithm is complex and generates multiple linked risk of recurrence (ROR) scores. The baseline ROR is derived from subtypes alone, ROR-P adds proliferation weighting to the subtype score whilst the ROR-PT score (applied in the clinical setting) uses tumour size weighting in addition to subtype and proliferation [6, 21]. For each given sample the algorithm, as described in the Prosigna patent (US20130337444A1), follows a series of steps: 1) Normalization of raw data to 8 housekeeping genes and log_2_-transformation. 2) Reference-sample-normalization of the data by dividing each gene by its values in the reference samples. (This reference sample is shipped as part of the Prosigna kit and was not relevant to code sets used for this study.) 3) Application of distinct centering and scaling factors to the normalized data from each of the 46 genes in the Prosigna algorithm resulting in each sample’s “row-scaled” data. These centering and scaling factors are not published and required estimation. 5) Calculation of the Pearson correlation between the sample’s row-scaled expression and the Prosigna centroids for the four subtypes. These centroids have been published, and were used *verbatim*. 6) Calculation of the proliferation score as the average row-scaled expression of the 18 proliferation genes. The identity of these 18 genes are published. 7) Calculation of the ROR-PT score as a linear combination of the 4 subtype’s Pearson correlations, proliferation score, and an indicator variable for tumor size > 2cm. The weights for this linear combination are also publicly available. 8) Linearly adjust ROR-PT to fall on roughly a 0–100 scale, and truncate any values falling outside this scale. The centering and scaling constants used for this rescaling are not publicly reported, and were estimated.

“Prosigna-*trained*” results (“ROR”, “ROR-P” & “ROR-PT”) were therefore calculated via recapitulation of the Prosigna algorithm with the following important deviations: 1) The reference sample normalization was not performed (step 2), 2) Centering factors for “row-scaling” are not published and were estimated, 3) Centering and scaling constants used for final rescaling for ROR-PT into a 0–100 range are not published and were estimated.

The derivation of the “Prosigna-*trained*” algorithm followed a two-step process:

Firstly we implemented the closest approximation to the Prosigna algorithm without training, following each step described above as follows. This differs from the approach in method 1 above in that it does not apply a global normalization to the data prior to applying the Prosigna algorithm but relies instead on normalization specific to the Prosigna algorithm. We normalized to the 8 housekeeping genes and for “row-scaling” making the assumption that each gene’s centering factor was its mean in our dataset, and each gene’s scaling factor was its SD in our dataset. Using the resulting row-scaled data, we performed the rest of the Prosigna algorithm consistent with the published approach (through step 6 in the above Prosigna summary, omitting the final mapping to a 0–100 scale).

Next we sought to improve on the above “untrained” fit by seeking centering and scaling factors that optimize the fit to true Prosigna ROR scores using the training dataset provided.

#### Procedure for optimizing centering and scaling factors given a training dataset

Starting with initial values of the centering and scaling factors equal to those used in the untrained algorithm we iterated between optimizing the scaling factors and optimizing the centering factors. Specifically, holding the centering factors constant, we took the scaling factors that achieve the best concordance between the true Prosigna-ROR-PT score and “Prosigna-*trained* ROR-PT” score. Then holding the scaling factors constant, we took the centering factors that optimized the Prosigna-ROR-PT vs. “Prosigna-*trained* ROR-PT” goodness of fit. As part of each optimization, we used linear regression to map “ROR-*trained*” scores to the 0–100 scale. Optimization was performed using the R function “optim” to minimize the mean squared error (MSE) between the Prosigna-ROR-PT and “Prosigna-*trained* ROR-PT” scores. Each iteration of the above process is expected to improve training set performance, but after a certain point these iterations may begin overfitting noise in the data and producing less accurate estimates.

**Fig 1:**
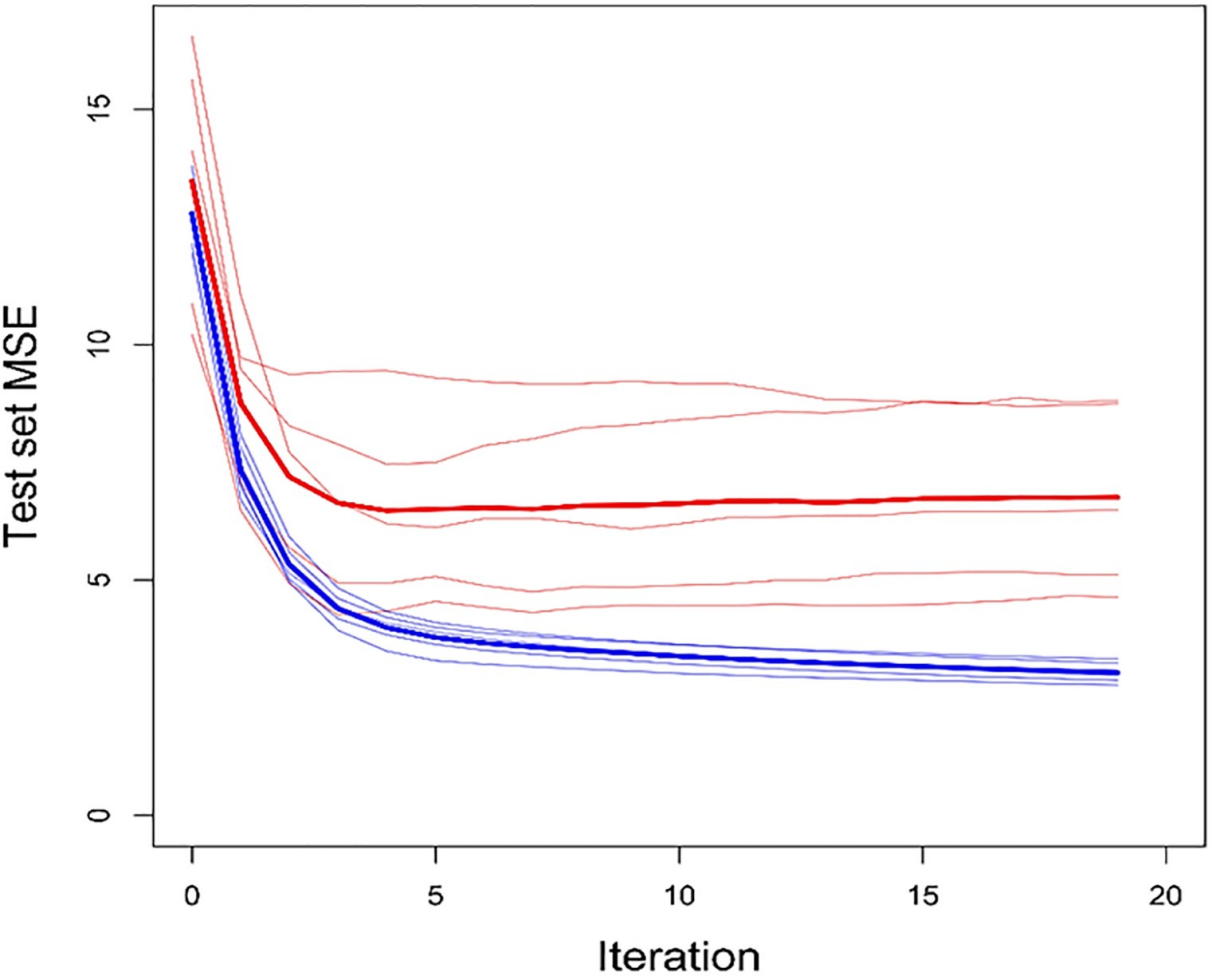
Training and test set performance over iterations of optimizing centering and scaling factors. Thin red lines track the 5 test sets’ performance; thick red line shows the average test set performance. Blue lines show training set performance. Performance is measured with Mean Squared Error (MSE) between ROR-trained scores and true ROR. MSE is calculated as the average MSE of ROR-PT and ROR-P scores, both of which are calculated with the same centering and scaling factors.

**Fig 2:**
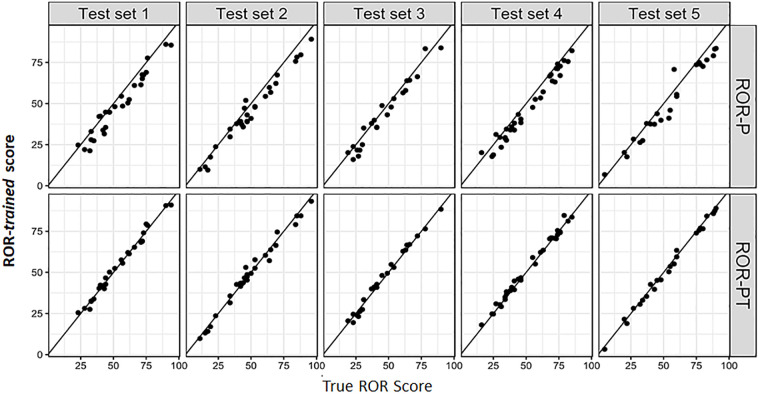
Prosigna ROR-P and ROR-PT training: Test set performance at selected iteration. For each of the 5 test sets, true ROR-PT and ROR-P is plotted against the corresponding “ROR-*trained*” score. Lines show the identity. Without any attempt at optimization, we achieve very high correlation (r > 0.98) between “ROR-*trained*” and true ROR scores. It appears that by taking centering and scaling factors from genes’ means and SDs in our data we get very close to the correct values.

To avoid overfitting noise in the data due to overtraining, we sought to restrict the number of rounds of iteration performed. We performed 5-fold cross-validation as follows: To choose the number of iterations of optimization to perform (the “tuning parameter”), we employed a cross-validation approach. We split the training data into 5 subsets and used each 1/5 subset as an independent test set, and used the 4/5 complement of each test set as a training set. We ran 20 iterations of optimization on each training set, and recorded algorithm performance on the corresponding test set. Based on the results of this cross-validation exercise, to train the final “Prosigna-*trained* ROR-PT” algorithms, we applied the same alternating optimization procedure to the complete training dataset, stopping after four iterations (Fig 1). The optimal performance across the 5 test sets occurred after the fourth iteration. Note that substantial test set performance improvements were gained over the untrained version (iteration 0). Fig 2 shows the test set performance in more detail at the chosen iteration for ROR-P and ROR-PT results.

#### b) Oncotype DX-*trained* scores

**Fig 3:**
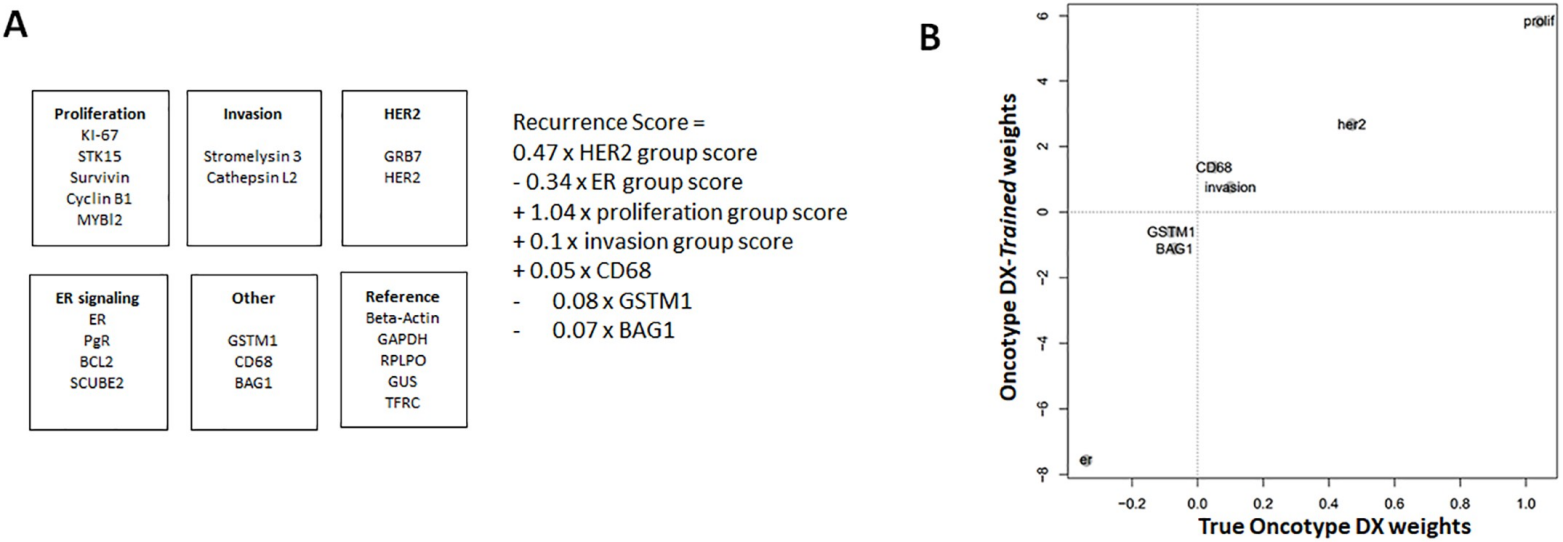
Oncotype DX training. Oncotype DX Recurrence Score metagenes and metagene weights (**A**) for Recurrence score calculation. (**B**) Oncotype DX metagene weights (x-axis) versus “Oncotype DX-*trained*” weights (y-axis) used to calculate “Oncotype DX-*trained* recurrence scores”.

The Oncotype DX algorithm for calculating recurrence scores (RS) represents a simple linear combination of score calculated for metagenes (5 genes for proliferation, 2 for *HER2*, 4 for *ER*, 2 for invasion and individual scores for *CD68*, *GSTM1*, and *BAG1*; Fig 3). In this setting we implemented the closest possible recapitulation of the Oncotype DX algorithm with the following departures. No sample provided *Cathepsin-L2* expression levels above background so this gene was excluded from algorithmic modelling. *GSTM1* showed a bimodal distribution with approximately half of all cases with expression levels below background, therefore this gene was thresholded at background. Finally the optimal fit for the metagenes in our training cohort was achieved by a slight modification of the metagene coefficient, predominantly with an increased weight of the ER metagene score.

#### c) MammaPrint-*trained* scores

We attempted to recapitulate the same approach used for the original MammaPrint algorithm but were unable to extract sufficient information from the description of the algorithm provided in relevant publications for us to achieve our objective. We therefore used the “ridge regression” machine learning technique to predict MammaPrint risk categories from the 70 genes used in the original algorithm. This approach was further compromised by the dichotomous nature of the MammaPrint results. We arrived at this method and chose a tuning parameter bases on a 5-fold cross validation approach within the OPTIMA prelim training set; the OPTIMA validation set was retained for an independent validation of the “MammaPrint-*trained*” scores against true MammaPrint scores.

## Results

### “Signature-*like*” versus “Signature-*trained*”: Accuracy and concordance *versus* true assay results

**Fig 4:**
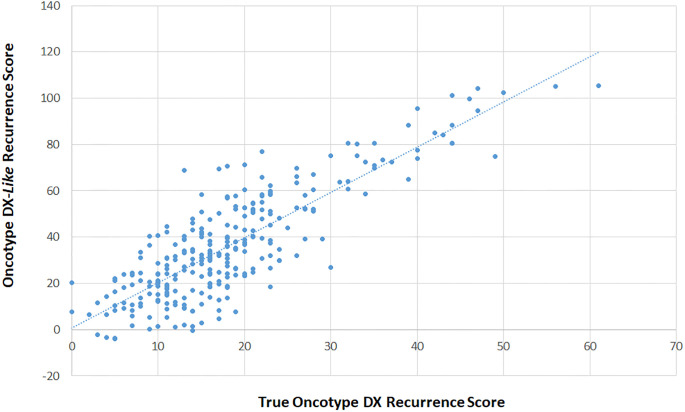
Comparison of “Oncotype-DX-*Like*” and true Oncotype-DX scores. Pearsons Correlation between “Oncotype DX-*Like*” scores calculated as described in method 1 (y-axis) and true Oncotype DX scores (x-axis) from the OPTIMA prelim study.

**Fig 5:**
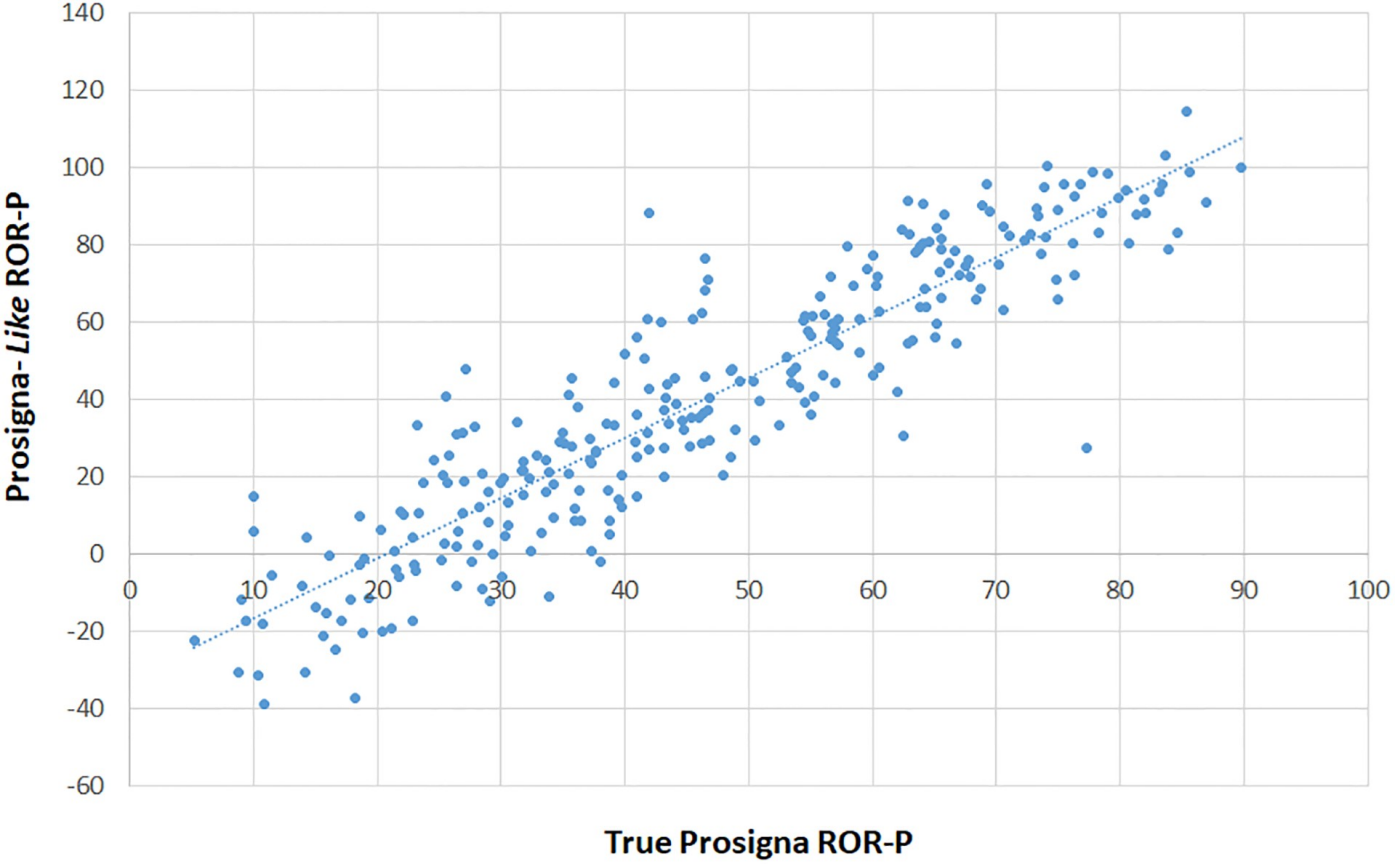
Comparison of Prosigna ROR true scores with “Prosigna-*like*”. Pearsons Correlation between “Prosigna-*like*” (ROR-P) scores calculated as described in method 1 (y-axis) and true Prosigna scores (x-axis) from the OPTIMA prelim study.

**Fig 6:**
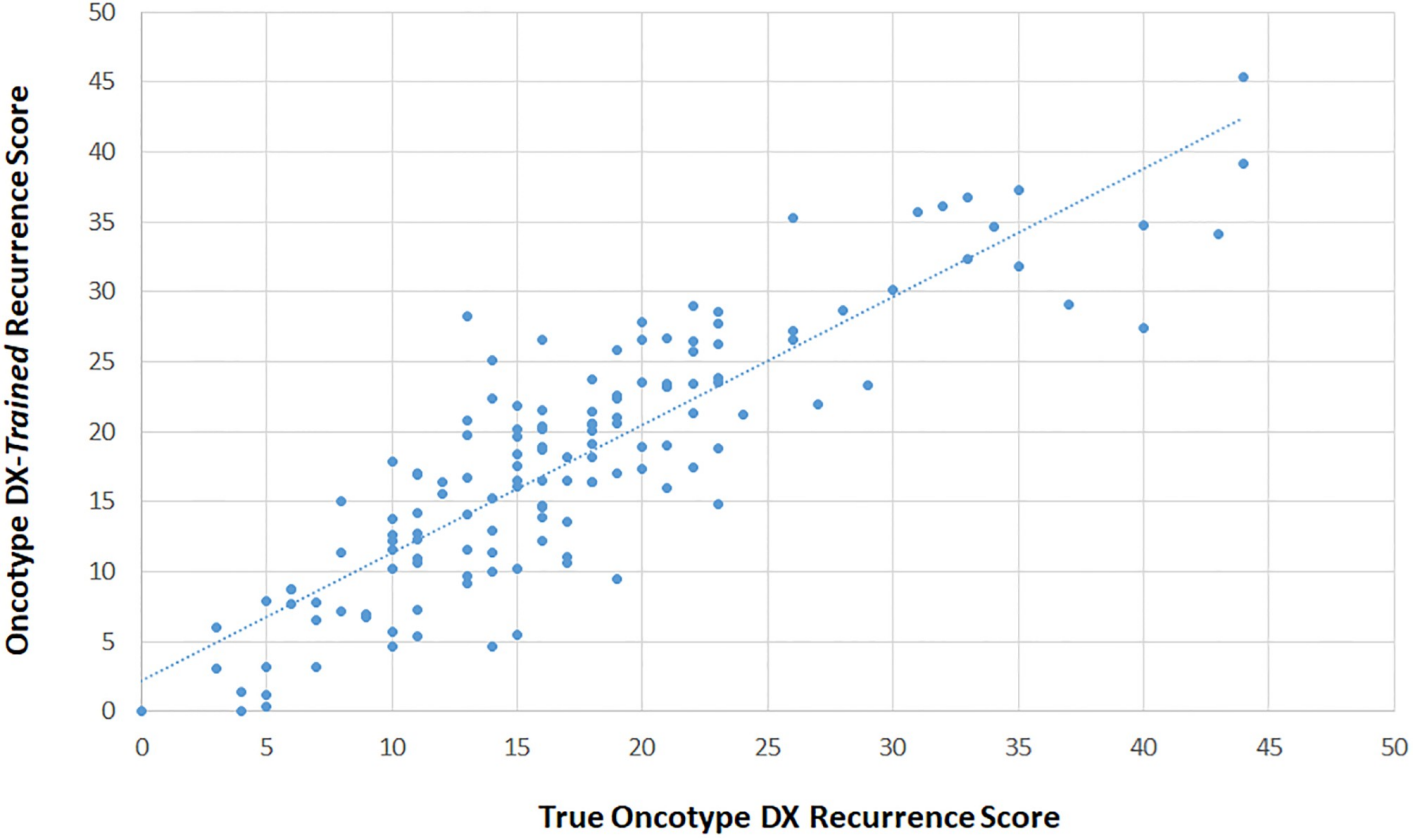
“Oncotype DX-*trained*” scores versus true Oncotype DX scores in the validation set only. Pearsons Correlation between “Oncotype DX-*trained*” scores calculated as described in method 1 (y-axis) and true Oncotype DX scores (x-axis) from the OPTIMA prelim study.

**Fig 7:**
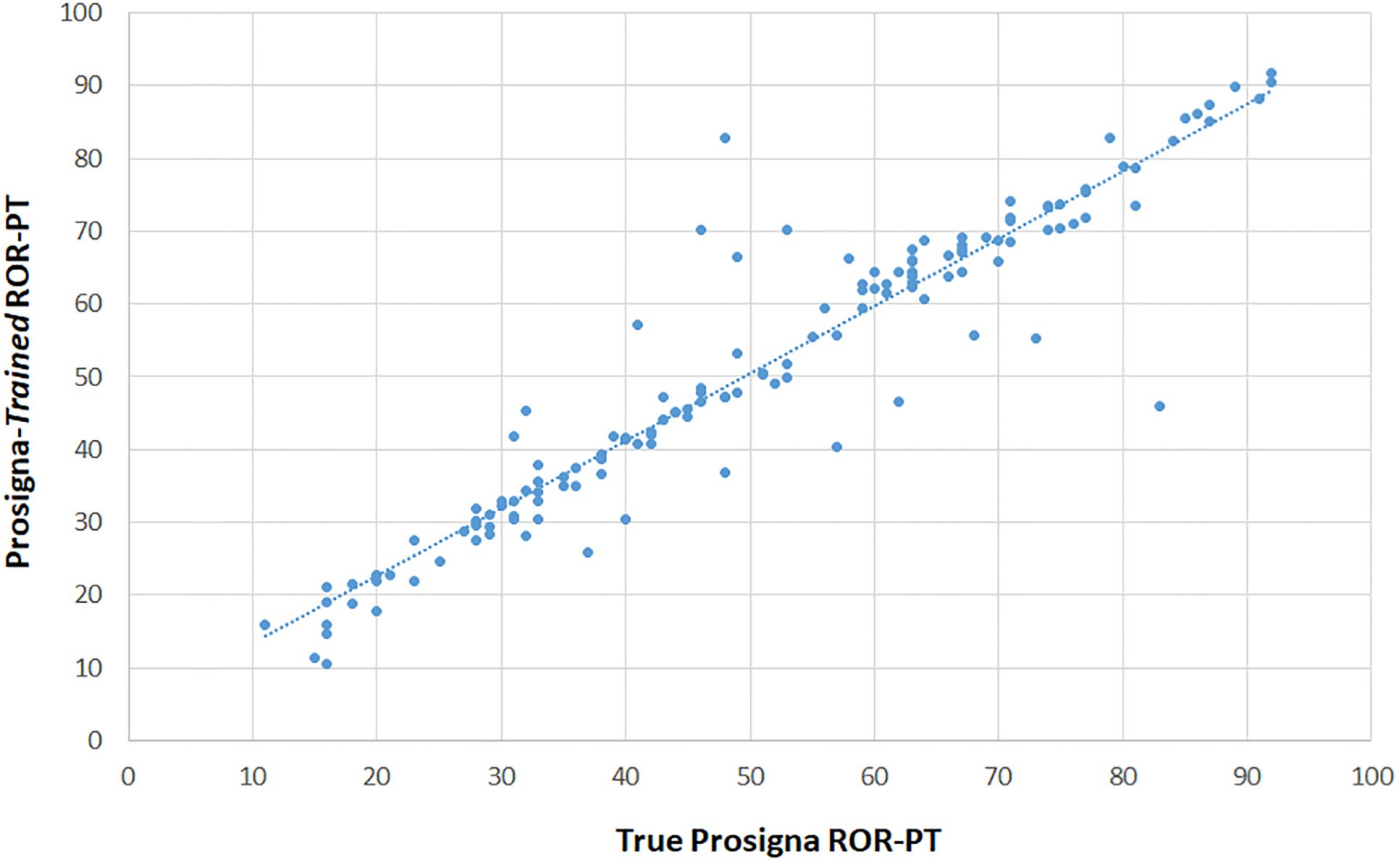
Comparison of “Prosigna-*Trained*” ROR-PT with true Prosigna ROR-PT in the validation set only. Pearsons Correlation between “Prosigna-*trained*” scores calculated as described in method 1 (y-axis) and true Prosigna scores (x-axis) from the OPTIMA prelim study.

For each of the computationally derived assay results from the modelling method (“signature-*like*”, e.g. “Oncotype DX-*like*” etc) and the training method (“signature-*trained*” e.g. “MammaPrint-*trained*” etc.) we performed simple regression analyses against either the full OPTIMA prelim cohort (“signature-*like*” risk scores) or the validation sets (“signature-*trained*” risk scores). The regression curves are provided in Figs 4–7 and data summarised in Table 1 and S1-S6 Tables in S1 File.

**Table 1:** Pearsons correlation or concordance between “true” risk signature results and “signature-*like*” or “signature-*trained”* results in the OPTIMA prelim dataset.

**Table 1a “signature like**				**All cases**			
				**Correlation coefficient**	**Slope**	**Intercept**	**Concordance**
Oncotype DX				0.873	1.95	1.26	
Prosigna				0.913	1.55	-31.19	
MammaPrint				NA	NA	NA	83.2%
**1B “signature trained”**	**Training set**			**Validation set**			
	**Correlation coefficient**	**Slope**	**Intercept**	**Correlation coefficient**	**Slope**	**Intercept**	**Concordance**
Oncotype DX	0.90	0.851	3.1521	0.87	0.9165	2.1496	
Prosigna	0.993	0.9929	0.3943	0.93	0.9261	4.86	
MammaPrint	NA	NA	NA	NA	NA	NA	81.7%

Correlations between true *versus* computed (“*like*” or “*trained*”) test results.

NA = not applicable.

Both “signature-*like*” (Pearsons R range 0.837–0.913) and “*signature-trained*” (0.870–0.940) scores showed highly significant correlations with the true results obtained from OPTIMA prelim. Overall, “signature-*trained*” scores showed closer agreement to true results both in terms of correlation and the slope (0.851–0.926) and intercept (2.15–4.86) of the regression equations comparing “*true*” versus “*trained*” results (Table 1; Figs 4–7; S1-S6 Tables in S1 File). Regression equations for “signature-*like*” results showed evidence of potential scaling effects, which compromised the ability of these results to provide the best estimate of real-world comparisons between tests (Table 1).

### Method 1: Comparison of true signature risk scores with “signature-*like*” risk scores

#### Comparison of Oncotype DX recurrence scores with Oncotype DX-*like* recurrence scores

Using a simple linear regression, we compared the true Oncotype DX Recurrence Score with the Oncotype DX-*like* Recurrence Scores (n = 274) generated as described in the methods above (Fig 4). The results showed a modest correlation (R = 0.837) with a regression equation suggesting the relationship between Oncotype DX-*like* recurrence scores and true Oncotype DX Recurrence scores is as follows:

“OncotypeDX-like”RecurrenceScore=1.26+1.95*OncotypeDXRecurrenceScore.

When grouped either into 3 (low, intermediate, high) or 2 (low *vs* high) risk groups based on the original and modified clinical groupings [2, 16] “Oncotype DX-*like*” scores correctly classified 42.3% and 54.7% of cases for ternary (as per OPTIMA prelim) and binary risk classification respectively. Only 2 cases were classified as lower risk using “Oncotype DX-*like*” scores versus true scores (using the ternary classification; S1a Table in S1 File), 56.9% and 45.2% of cases were assigned to higher risk groups when using ternary (S1a Table in S1 File) or binary (S1b Table in S1 File) groupings.

#### Comparison of true Prosigna ROR & ROR-P scores with Prosigna-*like* ROR & ROR-P scores

We applied a similar simple linear regression to compare the true Prosigna ROR-P scores with the Prosigna-*like* ROR-P scores (N = 274) generated as described above (Fig 5). The results showed a good correlation for ROR (R = 0.8996) and ROR-P (0.913) with regressions equation suggesting the relationships between Prosigna-*like* ROR-P scores and true ROR-P scores are as follows:

ROR-P:Prosigna-likeROR-Plikescore=−32.19+1.5*ProsignaROR-Ptruescores.

“Prosigna-*like*” scores correctly classified 86.1% & 86.9% of cases for ternary versus binary classification (S2a, S2b Tables in S1 File). Only 4 cases were classified as lower risk using “Prosigna-*like*” scores *versus* true scores (using ternary classification; S2 Table in S1 File), 27.0% and 11.7% of cases were assigned to higher risk groups when using ternary (S2a Table in S1 File) or binary (S2b Table in S1 File) groupings.

#### Comparison of MammaPrint scores with “MammaPrint-*like*” scores

No regression analysis could be performed using MammaPrint scores which are reported as a binary “Low” vs “High” risk. “MammaPrint-*like*” scores correctly classified 83.2% of cases when compared to true MammaPrint scores (S3 Table in S1 File). Roughly equal proportions of cases where scored lower (3.6%) or higher (4.4%) risk using “MammaPrint-*like*” versus true scores (S3 Table in S1 File).

### Method 2: Comparison of true signature risk scores with “signature-*trained*” risk scores

#### Comparison of Oncotype DX recurrence scores with “Oncotype DX-*trained*” recurrence scores

The “Oncotype DX-*trained*” recurrence scores achieved a high correlation with the true Oncotype DX recurrence scores from the validation cohort of the OPTIMA prelim study (Fig 6; right hand panel; S4 Table in S1 File). The relationship between “Oncotype DX-*trained*” recurrence scores and true Oncotype DX recurrence scores is represented by the equation:

“OncotypeDX-trained”RS=0.915xOncotypeDXTrueRS+2.15

The correlation coefficient in the validation cohort was 0.8700, marginally lower than in the training cohort (R = 0.9175).

When grouped into either 3 (low, intermediate, high) or 2 (low *vs* high) risk groups based on the original and modified clinical groupings [2, 16] “Oncotype DX-*trained*” scores correctly classified 75.2% of cases for the ternary and 90.1% for the binary classifications. Fewer cases (1.8% and 1.5%) were classified as lower risk using “Oncotype DX-*trained*” scores *versus* true scores whilst 19.0% and 8.0% of cases were assigned to higher risk groups when using ternary (S4a Table in S1 File) or binary (S4b Table in S1 File) groupings.

#### Comparison of Prosigna ROR-PT scores with “Prosigna-*trained*” ROR-PT scores

The “Prosigna-*trained*” ROR-PT algorithms scores were compared to true Prosigna scores in the reserved validation set of the OPTIMA prelim data. Most true scores were predicted with very high accuracy, with a small number of samples returning discordant scores (Fig 7; S5 Table in S1 File). The results showed excellent correlation (0.9472) with a regression equation suggesting the relationships between Prosigna-*trained* ROR-PT scores and true ROR-PT scores are as follows:

Prosigna-trainedROR-PTscore=4.10+0.9261*ProsignaRORPtruescores.

“Prosigna-*trained*” ROR-PT scores correctly classified 89.8% and 95.3% of cases for ternary versus binary classification (S5 Table in S1 File). Roughly equal proportions of cases were scored lower (5.5% & 1.8%) or higher (4.7% & 2.9%) risk using ternary or binary “Prosigna-*trained*” ROR-PT versus true scores (S5 Table in S1 File).

#### Comparison of MammaPrint scores with “MammaPrint-*trained*” scores

No regression analysis could be performed using MammaPrint scores which are reported as a binary “Low” vs “High” risk. “MammaPrint-*trained*” scores correctly classified 90.5% of cases when compared to true MammaPrint scores (S6 Table in S1 File).

## Discussion

The goal of the current study was to explore the potential to use surrogate gene signatures to provide robust information on the impact of discordant risk classification by different molecular prognostic signatures in early breast cancer. Since the implementation of prognostic assays, using gene expression profiling, multiple studies have demonstrated the benefit of this approach particularly for ER+ve breast cancers treated with curative intent [3, 6, 12, 13, 16, 20–25]. However, as demonstrated by us, and others, there are a number of challenges relating to these prognostic assays and in particular for patients, clinicians and health care providers who are seeking to gain the maximum potential information from the plethora of such assays now available.

With multiple potential tests available, there is a significant lack of real world data which allows comparison between such tests at the individual patient level. Such data requires either analysis of large cohorts of patients with commercial assays, an approach which is both cost prohibitive and unlikely to secure support from multiple commercial entities, or an analysis using surrogate methods to estimate the impact of “real world” assays. Such approaches have been reported previously, by us [4] and others [20, 24–26], but interpretation of these results has been hampered by concerns as to the accuracy of such methods in recapitulating true assay results.

A second challenge is clear evidence that combining prognostic assays, particularly adding conventional histopathology information to assay readout, provide additional prognostic information over and above any assay applied in isolation [12]. Moreover, the prognostic information provided by any individual assay may, in fact, be relatively modest [14]. To address this challenge, several studies have sought to either directly compare different commercial tests [12] or to recapitulate test results from global transcriptome data [26]. By using a curated data set from the OPTIMA prelim study we have been able to demonstrate the accuracy with which two different informatics approaches can mirror real world assay results across 3 commonly used prognostic tests in breast cancer.

Both approaches achieved highly significant correlations between estimated and true results with Pearson coefficients between 0.837–0.913 for “signature-*like*” and 0.870–0.940 for “signature-*trained*” results. However, regression equations for “signature-*like*” scores showed evidence of potential scaling effects, whilst those from “signature-*trained*” scores were more closely fitted to the true results, when the slope and intercept of the regression lines were taken into consideration. Overall, estimating assay results solely using the published algorithms from each assay resulted in lower concordance between “signature-*like*” and true assay results (42.3–86.1%) than comparisons between “signature-*trained*” and true assay results (75.2–90.5%) when ternary groupings were used for each assay. For binary categorization, as now used for chemotherapy selection and based on the cutpoints used for the TAILORx trial [2], concordance rates were 54.7–86.9% for “signature-*like*” and 90.1–95.3% for “signature-*trained*” results. There is therefore clear evidence, across multiple metrics, that the training approach, as would be expected, is superior to methods using assay algorithms alone without reference to true assay results.

Overall, “signature-*like*” results lead to an over-estimation of risk more often than true results for the majority of cases with discordant results for both the Oncotype DX and Prosigna assays (S1 and S2 Tables in S1 File), whilst for MammaPrint this effect was less obvious (S3 Table in S1 File). Training produced markedly fewer discordant cases, as predicted, but whilst the effect for the Prosigna assay appeared more balanced (S4 Table in S1 File), for the Oncotype DX assay the majority of misclassified cases after training still appeared to reflect an over-estimation of risk (S5 Table in S1 File).

In summary, we provide perhaps for the first time, a comparison between prognostic risk scores estimated by computational analysis of expression profiles and true assay results. As predicted, using a “training and validation” approach to compute risk scores provides a closer fit to true results than the more widely used approach where results are calculated based on published test algorithms. High degrees of concordance between “*trained*” and actual assay scores, both as continuous variables and as a categorical readout, suggest that this approach can be applied to large cohorts and provide information estimating “real world” performances of different assays. Untrained models (i.e. “signature-*like*”) may, as seen in this case, be more challenging to interpret. We conclude that our “signature-*trained*” results provided results that closely reflect the performance of the true assays and provide the basis for further examination of large-scale gene expression datasets in which it would not be feasible to perform multiparametric testing using original methodology.

## Supporting information

S1 File (DOCX)
