# Supplementary material for: Computational approaches to support comparative analysis of multiparametric tests: Modelling versus Training
Source: PLoS One. 2020 Sep 3;15(9):e0238593. doi: 10.1371/journal.pone.0238593 (PMC7470374; doi:10.1371/journal.pone.0238593)
Supplement: S1 File — (DOCX) [file pone.0238593.s001.docx]

**Supplementary Tables:**

**S1 Table: Cross tabulation between “Oncotype DX-*like*” and true Oncotype DX scores by Risk Category. a)** Cross tabulation for “Oncotype DX-*like*” and true Oncotype DX scores categorized as low (0-18), intermediate (18-25) and high risk (>25), as per original OPTIMA prelim); **b)** or Low (0-25) versus high risk (>25) according to TAILORx binary classification.

| **S1a** |  | **“Oncotype DX-*like*”** | | |
| --- | --- | --- | --- | --- |
| N= 274 |  | **Low** | **Intermediate** | **High** |
| True- Oncotype DX | Low | **57** | 57 | 52 |
|  | Intermediate | 1 | **13** | 47 |
|  | High | 0 | 1 | **46** |

| **S1b** |  | **“Oncotype DX-*like*”** | |
| --- | --- | --- | --- |
| N= 274 |  | **Low** | **High** |
| True- Oncotype DX | Low | **103** | 124 |
|  | High | 0 | **47** |

**S2 Table: Cross tabulation between “Prosigna-*like*” and true Prosigna scores by Risk Category. a)** Cross tabulation for “Prosigna-*like*” and true Prosigna scores categorized as low (0-40), intermediate (40-60) and high risk (>60); **b)** or Low (0-60) versus high risk (>60) in line with TAILORx binary classification.

| **S2a** |  | **“Prosigna-*like*”** | | |
| --- | --- | --- | --- | --- |
| N= 274 |  | **Low** | **Intermediate** | **High** |
| True Prosigna | Low | **64** | 52 | 0 |
|  | Intermediate | 0 | **49** | 32 |
|  | High | 0 | 4 | **73** |

| **S2b** |  | **“Prosigna-*like*”** | |
| --- | --- | --- | --- |
| N= 274 |  | **Low** | **High** |
| True Prosigna | Low | **165** | 32 |
|  | High | 4 | **73** |

**S3 Table: Cross tabulation between “MammaPrint-*like*” and true MammaPrint scores.** In line with MammaPrint reporting only binary categorisation is shown.

|  |  | **“MammaPrint-*like*”** | |
| --- | --- | --- | --- |
| N= 274 |  | **Low** | **High** |
| True MammaPrint | Low | **131** | 12 |
|  | High | 10 | **97** |

**S4 Table: Cross tabulation between “Oncotype DX-*trained*” and true Oncotype DX scores by Risk Category. a)** Cross tabulation for “Oncotype DX-*trained*” and true Oncotype DX scores categorized as low (0-18), intermediate (18-25) and high risk (>25, as per original OPTIMA prelim); **b)** or Low (0-25) versus high risk (>25) according to TAILORx binary classification.

| **S4a** |  | **“Oncotype DX-*trained*”** | | |
| --- | --- | --- | --- | --- |
| N= 274 |  | **Low** | **Intermediate** | **High** |
| True- Oncotype DX | Low | **134** | 30 | 2 |
|  | Intermediate | 0 | **30** | 20 |
|  | High | 1 | 4 | **42** |

| **S4b** |  | **“Oncotype DX-*trained*”** | |
| --- | --- | --- | --- |
| N= 274 |  | **Low** | **High** |
| True- Oncotype DX | Low | **205** | 22 |
|  | High | 4 | **42** |

**S5 Table: Cross tabulation between “Prosigna-*trained*” and true Prosigna scores by risk category. a)** Cross tabulation for “Prosigna-*trained*” and true Prosigna scores categorized as low (0-40), intermediate (40-60) and high risk (>60); **b)** or Low (0-60) versus high risk (>60) in line with TAILORx binary classification.

| **S5a** |  | **“Prosigna-*trained*”** | | |
| --- | --- | --- | --- | --- |
| N= 274 |  | **Low** | **Intermediate** | **High** |
| True Prosigna | Low | **111** | 5 | 0 |
|  | Intermediate | 10 | **63** | 8 |
|  | High | 0 | 5 | **72** |

| **S5b** |  | **“Prosigna-trained”** | |
| --- | --- | --- | --- |
| N= 274 |  | **Low** | **High** |
| True Prosigna | Low | **189** | 8 |
|  | High | 5 | **72** |

**S6 Table: Cross tabulation between “MammaPrint-*trained*” and true MammaPrint scores.** In line with MammaPrint reporting only binary categorisation is shown.

|  |  | **“MammaPrint-trained”** | |
| --- | --- | --- | --- |
| N= 274 |  | **Low** | **High** |
| True MammaPrint | Low | **146** | 21 |
|  | High | 5 | **102** |
